# Supplementary material for: Rapid turnover of life-cycle-related genes in the brown algae
Source: Genome Biol. 2019 Feb 14;20:35. doi: 10.1186/s13059-019-1630-6 (PMC6374913; doi:10.1186/s13059-019-1630-6)
Supplement: Supplementary file 3 — Protein alignments, corresponding Gblocks html file and CDS sequences for the lineage conserved gametophyte-biased genes. (ZIP 2109 kb) [file 13059_2019_1630_MOESM3_ESM.zip › Con_GA_alignments/Ec-11_004170.aa_ali.fasta-gb.htm]

Ec-11\_004170.aa.fasta.trans.aa\_ali.fasta


## Gblocks 0.91b Results

Processed file: **Ec-11\_004170.aa.fasta.trans.aa\_ali.fasta**  
Number of sequences: **2**  
Alignment assumed to be: **Protein**  
New number of positions: **709** (selected positions are underlined in blue)

```
                         10        20        30        40        50        60
                 =========+=========+=========+=========+=========+=========+
Ec-11_004170     MSNQPQYYRITDSLESLNVRIRLRKIFGRSINTNTGAVGKVRPGKKGDHGDDVDNAPGSF
MSTRG.53257.1    ------------------------------------------------------------
                                                                             


                         70        80        90       100       110       120
                 =========+=========+=========+=========+=========+=========+
Ec-11_004170     LPGDEADIRVKWQEKVFGPEDVVTLSSGQRRGGKSQQQRAVGPPDNALSRLERDYAGHLE
MSTRG.53257.1    ------------------------------------------------------------
                                                                             


                        130       140       150       160       170       180
                 =========+=========+=========+=========+=========+=========+
Ec-11_004170     DLRRDGVDPLESLSEVMLFTYTDKDGFVPPSDKAGAA-----EGGLTTGDGGPFSCLEEA
MSTRG.53257.1    ----------------MLFTYTDKDGFVPPSSNTSRGDGSGGGGGLTTGDRGTLSYLEEA
                                 ###############            #################


                        190       200       210       220       230       240
                 =========+=========+=========+=========+=========+=========+
Ec-11_004170     VTSMPRTVASSLLETAKTAAGGGRGAGGGGASGRNSRAGVSDTAAVIQAHLAREDKYKVM
MSTRG.53257.1    VTNMPR-------KTATSSPDGTVASGGGGLTVGINRTGVSETAAVIQTHLAREDKFKVM
                 ######        ##############################################


                        250       260       270       280       290       300
                 =========+=========+=========+=========+=========+=========+
Ec-11_004170     YIMAAVDVDVREATKVAKSRAAAPGGGSRWRQGEDGV-GIHAHEQVVCSIKAHQNGTLEI
MSTRG.53257.1    YIMAAVDVDVRGAKKEAKIRANSPGG-ARWKRGGDGAGGVHLHEQVVCSIKAHQNGTLEI
                 ##########################            ######################


                        310       320       330       340       350       360
                 =========+=========+=========+=========+=========+=========+
Ec-11_004170     APGFSAEEPESGDEGAFLTDHTLKVMDKEGARLTTYRFTSPSGGVFSYTVENDSEPGGGV
MSTRG.53257.1    APGFSAEEPESGDEGAFLTDHTLKVMDKEGARLTTYRFTSPSGGVFSYTVENDSEPGGGV
                 ############################################################


                        370       380       390       400       410       420
                 =========+=========+=========+=========+=========+=========+
Ec-11_004170     EEIEAWEAEEASRDLWKKASRQQNAGTQLVGAPVAGGAVALHVFMEIVSASGFAGVDRLY
MSTRG.53257.1    EEMEAWEAEEASRDLWKKASRQQTAGGQLLGAPAAGGAVALHAFMEIVSASGFTGVDRLY
                 ############################################################


                        430       440       450       460       470       480
                 =========+=========+=========+=========+=========+=========+
Ec-11_004170     TEYALCLPRHWTMADEEGSRGAGGGGGQHGSGRGDRGDHTFAWENGGNGDGSGDFAAGGT
MSTRG.53257.1    TEYGLCLPRTWSMAESEG--GASGERGENNDGR----DEGFVEGRGRATREHGEFAAAGT
                 ##################  #############    #######################


                        490       500       510       520       530       540
                 =========+=========+=========+=========+=========+=========+
Ec-11_004170     TQVASQAYPLAVPHRSVRSATTTTTRKSDTRSGLGHGTSSASVTTLSSSSTINTYGNSAM
MSTRG.53257.1    TQVACQDFPLAVPHRSVKFDGTT--SKSHASGNLGHAASFSSPLAPSPAG-ASMYGSSTM
                 #######################   #####################       ######


                        550       560       570       580       590       600
                 =========+=========+=========+=========+=========+=========+
Ec-11_004170     PAAFQAPPKIVMSGAALRATSAILLCFLGLLASLGESPLFWVVLGGAVVVVAAITGGGSG
MSTRG.53257.1    PTAFQAPPRTAASGATLRVGSAVLLCFLGLLGSLGESPLLWVVFGGAVVVVAALSGDGGY
                 ##########################################################  


                        610       620       630       640       650       660
                 =========+=========+=========+=========+=========+=========+
Ec-11_004170     ----GGEGGSPVAHFGFPAEFHLINEEEGEMAGIGERPQILLAVSSLDGLARHRVEGYGY
MSTRG.53257.1    GGWERGEGGVPVAHFGFPAEFHLFNDEEGETAGLGERPQVLLAVSSLDSLARHRVEGYGY
                      #######################################################


                        670       680       690       700       710       720
                 =========+=========+=========+=========+=========+=========+
Ec-11_004170     VSFPRQPGAHELHIRTWRPVCSIRTHLQDFFVGGAHRLHDPRYASKPGSFRGTFLSRFGF
MSTRG.53257.1    LSFPRQAGAHELFIRTWRPVCSLRTHLQDFFVGGAHRLHDPRYASKPGGFTGTFLNRFGF
                 ############################################################


                        730       740       750       760       770       780
                 =========+=========+=========+=========+=========+=========+
Ec-11_004170     RTETSGTLRVRLHLAEQGRVRDLRAESGGEGGGPTIDLAGTRTVKEILRVVNAKRMGVEI
MSTRG.53257.1    RTETSGTLRVRLQLAEQGRVCDLQAVPGAAGVGPGIDLAGTRTVKEILRMVNAKRMGTEI
                 ############################################################


                        790       800       810       820       830       840
                 =========+=========+=========+=========+=========+=========+
Ec-11_004170     GRRFSATVDPASPSAAAAPTTPAAGSAAPAAAPAASS---TAKPREGVGGGRGMGGVSGG
MSTRG.53257.1    GRRFSATAESTSP-AATHPTTTSGSAAPAAAAPAASSGIGTARHKGNASRGTAAKDCSDT
                 ############# #######################   ####################


                        850       860       870       880       890       900
                 =========+=========+=========+=========+=========+=========+
Ec-11_004170     GVSHDEDNATIPLLGSRNPFERTLG-GKGTGKIEAAAHLLATVRGQLERSEQRTKGSLVE
MSTRG.53257.1    PLSSG-HNASMPLLGSRNPFEKTVEGGARTEKVKAATDLLARVRGQLERSEQRTKGLLVE
                 ###    ################   ##################################


                        910
                 =========+=
Ec-11_004170     RGGAGDMAPPR
MSTRG.53257.1    GRGVSDIAPTR
                 ###########
```

```
Parameters used
Minimum Number Of Sequences For A Conserved Position: 2
Minimum Number Of Sequences For A Flanking Position: 2
Maximum Number Of Contiguous Nonconserved Positions: 8
Minimum Length Of A Block: 10
Allowed Gap Positions: None
Use Similarity Matrices: Yes
```

```
Flank positions of the 13 selected block(s)
Flanks: [137  151]  [164  186]  [195  266]  [279  438]  [441  453]  [458  503]  [507  527]  [535  598]  [606  793]  [795  817]  [821  843]  [848  863]  [867  911]  

New number of positions in Ec-11_004170.aa.fasta.trans.aa_ali.fasta-gb:  709  (77% of the original 911 positions)
```
